# Stress Inducible Expression of *AtDREB1A* Transcription Factor in Transgenic Peanut (*Arachis hypogaea* L.) Conferred Tolerance to Soil-Moisture Deficit Stress

**DOI:** 10.3389/fpls.2016.00935

**Published:** 2016-06-28

**Authors:** Tanmoy Sarkar, Radhakrishnan Thankappan, Abhay Kumar, Gyan P. Mishra, Jentilal R. Dobaria

**Affiliations:** Crop Improvement Division, ICAR-Directorate of Groundnut ResearchJunagadh, India

**Keywords:** abiotic stress tolerance, drought stress, growth-related traits, groundnut, genetic transformation, regeneration

## Abstract

Peanut, an important oilseed crop, is gaining priority for the development of drought tolerant genotypes in recent times, since the area under drought is constantly on the rise. To achieve this, one of the important strategies is to genetically engineer the ruling peanut varieties using transcription factor regulating the expression of several downstream, abiotic-stress responsive gene(s). In this study, eight independent transgenic peanut (cv. GG20) lines were developed using *AtDREB1A* gene, encoding for a transcription factor, through *Agrobacterium*-mediated genetic transformation. The transgene insertion was confirmed in (T_0_) using PCR and Dot-blot analysis, while copy-number(s) was ascertained using Southern-blot analysis. The inheritance of *AtDREB1A* gene in individual transgenic plants (T_1_ and T_2_) was confirmed using PCR. In homozygous transgenic plants (T_2_), under soil-moisture deficit stress, elevated level of *AtDREB1A* transgene expression was observed by RT-PCR assay. The transgenic plants at 45-d or reproductive growth stage showed tolerance to severe soil-moisture deficit stress. Physio-biochemical parameters such as proline content, osmotic potential, relative water content, electrolytic leakage, and total-chlorophyll content were found positively correlated with growth-related traits without any morphological abnormality, when compared to wild-type. qPCR analysis revealed consistent increase in expression of *AtDREB1A* gene under progressive soil-moisture deficit stress in two homozygous transgenic plants. The transgene expression showed significant correlation with improved physio-biochemical traits. The improvement of drought-stress tolerance in combination with improved growth-related traits is very essential criterion for a premium peanut cultivar like GG20, so that marginal farmers of India can incur the economic benefits during seasonal drought and water scarcity.

## Introduction

Peanut or groundnut (*Arachis hypogaea* L.) is an important, leguminous oilseed crop, which is cultivated mostly in the tropical and sub-tropical regions of the world, under rainfed environments and low-input agricultural system (Bhauso et al., 2014b). Across the globe, its cultivation occupies ~20–25 million ha of land, yielding 35–40 million tons of pods annually. India stood second in peanut production after China, having ~5–6 million ha of cultivated area and 6–7 million tons of production (Bhauso et al., 2014a; Mishra et al., 2015). Its productivity suffers from recurrent drought due to insufficient, untimely, and erratic rainfall in those agro-climate areas where it is cultivated (Bosamia et al., 2015). Globally, about 20% of the land surface is under drought at any point of time (Burke et al., 2006); thus, considered as the major environmental constraint to productivity. Annually, peanut productivity suffers a loss of around 6 million tons due to drought alone, in different parts of the World (Bhatnagar-Mathur et al., 2014).

Till now, only minor QTLs were identified for water use efficiency (WUE) in peanut, moreover association of these QTLs with other trait is still unknown. Therefore, introgression of such QTLs, using marker assisted selection (MAS) may result in linkage drag (Bhatnagar-Mathur et al., 2008, 2014). Further, introgression of traits from diploid- or wild- specie to cultivated peanut is very difficult due to cross incompatibilities (Holbrook et al., 2011), linkage-drag (Tiwari et al., 2008), and time consuming breeding practices (Mishra et al., 2015). Thus, genetic engineering of peanut using heterologus drought-tolerance gene, under the control of stress inducible promoter, could be a prudent option to address this problem.

In order to restore cellular function and make plant more tolerant to multiple abiotic stresses, transferring of a single action gene may not be sufficient to confer the required level of tolerance (Bhatnagar-Mathur et al., 2008; Brasileiro et al., 2014). Thus, for enhancing the abiotic stress tolerance like drought, it is more appropriate to transfer a single-gene, encoding for transcription factors (TFs). The TFs regulate the expression of stress inducible, multiple downstream genes through signal transduction pathways, which in turn modulate a series of changes in the plant for adaptation (Datta et al., 2012; Anbazhagan et al., 2015).

The dehydration responsive element binding proteins (DREBs) are important TFs which comprise of conserved ERF/AP2 DNA-binding domain and regulate expression of a set of abiotic stress inducible, downstream, endogenous genes by binding to *cis* acting element (DRE/CRT) of their promoter and impart abiotic-stress tolerance (Pruthvi et al., 2014). The *DREB* transcription factors could be grouped into *DREB1*/*CBF* (CRT binding factor) and *DREB2*, which are involved in two separate signal transduction pathways under low temperature; salt and dehydration respectively (Shinozaki and Yamaguchi-Shinozaki, 2000; Pandey et al., 2014). *AtDREB1A* transgenic peanut genotype, showed tolerance to soil-moisture deficit stress, developed using cv. JL24 (Bhatnagar-Mathur et al., 2007); a variety which is out of cultivation in India. However, overexpressing *AtDREB1A* gene in transgenic peanut under the control of *rd29A* promoter showed tolerance to water-deficit stress with improved physiological and agronomic traits such as transpiration efficiency (TE) (Vadez et al., 2007); root-traits (Vadez et al., 2013); higher yield; and improved harvest-index (HI) (Bhatnagar-Mathur et al., 2014).

Constitutive co-expression of three stress-responsive TFs (*AtDREB2A, AtHB7*, and *AtABF3*) in transgenic peanut conferred tolerance, and produced improved total biomass under various abiotic stresses (Pruthvi et al., 2014). However, it could be better option to genetically engineer peanut by stress inducible expression of single TF such as *AtDREB1A* to achieve the desired level of stress tolerance and yield-gain under drought stress, thereby minimizing excessive metabolic load of the transgenics. An earlier report from our lab on the *AtDREB1A* transgenic peanut, revealed insights about the physio-biochemical parameters and its relationship with the improvement in productivity along with root-traits under both drought- and salinity- stresses (Sarkar et al., 2014). Furthermore, the present study illustrates the development of *AtDREB1A* transgenic lines and the relationship between transgene expressions with the improved physio-biochemical traits in transgenics, at reproductive-stage, under progressive soil-moisture deficit (drought) stress.

The ultimate aim of this investigation is to develop transgenic peanut cv. GG20; a high yielding, premium, bold-seeded, Virginia bunch type variety; most popular in Western part of India; for improved tolerance to soil-moisture deficit stress, by expressing *AtDREB1A* gene under the control of *rd29A* promoter, and to identify best transgenic plants with tolerance to terminal drought.

## Materials and methods

### Gene construct

The *Agrobacterium tumefaciens* strain GV3101 harboring binary vector pCAMBIA2300 containing P*rd29A*:*AtDREB1A*:3′ *nos* and P*CaMV35S*:*npt*II:35S *poly*A expression cassettes, obtained from IARI, New Delhi (India) was used for the genetic transformation. The binary vector comprised of kanamycin resistance gene for bacterial selection (Hajdukiewicz et al., 1994) and harbored *npt*II gene for selection of genetically transformed plant cells. The *npt*II expression cassette was fused with transgene expression cassette, isolated from pBI121 binary vector, containing *AtDREB1A/CBF3* cDNA (GenBank accession no. AB007787.1) driven by stress inducible *rd29A* promoter of *Arabidopsis thaliana* (GenBank accession no. D13044) and *nos* terminator.

*Agrobacterium* suspension culture was prepared by inoculating a single bacterial colony in liquid YEM medium (50 mL) containing 25 mgL^−1^ each of kanamycin, gentamycin, and rifampicin and grown overnight in a rotary shaker (120 rpm; 28°C). The cultures (OD_600_ = 0.6) were then harvested by centrifugation (5000 × g for 10 min) at room temperature (RT), and cells were re-suspended in 50 mL of co-cultivation MS medium (liquid) (Murashige and Skoog, 1962).

### Plant materials

The seeds of peanut cv. GG20 were obtained from the Genetic Resources Section of the Directorate of Groundnut Research (DGR), Junagadh, India.

### *Agrobacterium* mediated transformation and regeneration of transgenic peanut

**Figure 1:**
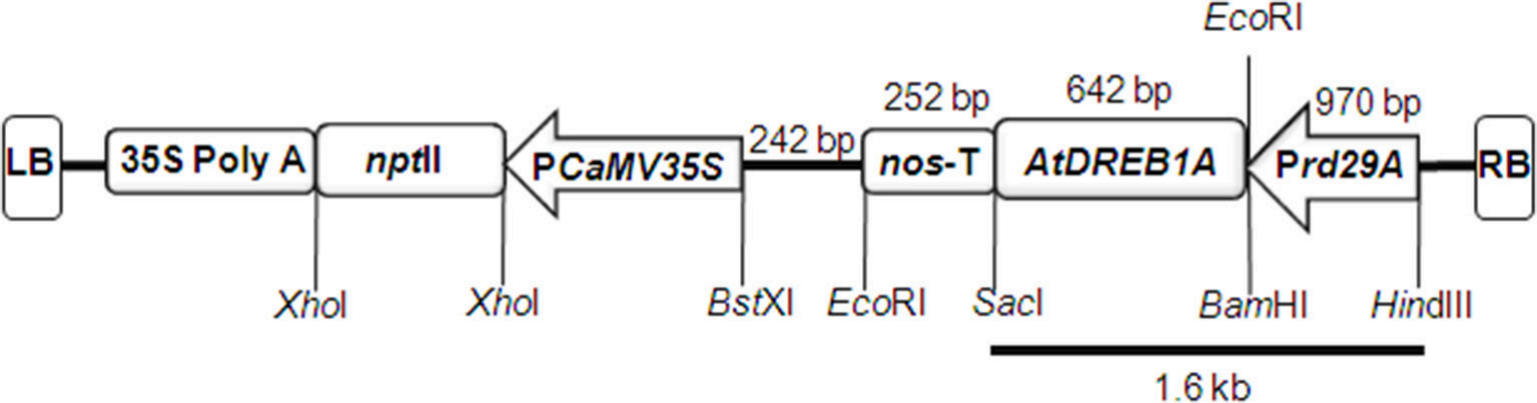
**A diagrammatic representation of pCAMBIA2300 plant transformation vector used for transforming tobacco and peanut explants**. The vector is harboring *rd29A* promoter, *AtDREB1A* transgene, and 3′ *nos* terminator expression cassette between *Sac*I and *BamH*I cloning sites.

Mature peanut seeds were surface sterilized in 70% alcohol for 30 s, and then in 0.1% mercuric chloride solution for 4 min, followed by six times rinsing using Milli-Q water and 15 min soaking in Milli-Q water. The embryo was then removed and cotyledonary explants were allowed to get infected with suspension culture of *A. tumefaciens* harboring binary vector (Figure 1) for 20 min at RT. The genetic transformation protocol as proposed by Mehta et al. (2013) was used for the regeneration of genetically transformed shoots, after which kanamycin based selection of putatively-transformed shoots was done (Bhatnagar-Mathur et al., 2007).

All the cultures were maintained at 26 ± 2°C in 88 μmol photon m^−2^s^−1^ light intensity using white fluorescent tube-lights, under 12 h light and 12 h dark photoperiod in a culture room throughout the experiment. Putative transgenic plants were hardened and allowed to set seeds under controlled polyhouse facility with set humidity (45%) and temperature (30 ± 2°C).

### DNA extraction and screening of transgenic plant by PCR

Genomic DNA was extracted from healthy young leaves of putative peanut transgenics using CTAB method (Doyle and Doyle, 1987), and quantified using Nanodrop spectrophotometer (ND-1000, Thermo Scientific, USA). PCR was performed using thermal cycler (Eppendorf, GmbH, Germany) to screen the putative transgenics for the presence of transgene using *AtDREB1A* gene specific primer pair (F:5-GCT CCG ATT ACG AGT CTT CGG-3′; R:5′-CTT CTG CCA TAT TAG CCA AC-3′) amplifying 528 bp product. The PCR was carried in 25 μl reaction volume containing 1 × PCR buffer, 2 mM MgCl_2_, 1.6 μL of dNTP mix (2 mM each), 25 pmole of each primers (Forward and Reverse), 1 U *Taq* DNA polymerase (Ferments, USA), and 200 ng of genomic DNA as template. A touch down PCR amplification profile was programmed at 94°C for 3 min for a initial denaturation, followed by first 8 cycles of 94°C for 45 s, 63–55°C for 45 s, and 72°C for 1 min, with a 1°C decrement in annealing temperature per cycle; then 27 cycles of 94°C for 45 s with constant annealing temperature of 55°C for 45 s and 72°C for 1 min followed by a final extension for 5 min at 72°C. The genomic DNA of *AtDREB1A* transgenic tobacco and plasmid DNA were used as positive control, while that of wild-type (WT) or genetically non-transformed peanut plants as negative control. PCR products were analyzed using gel electrophoresis in 1.2% (w/v) agarose gels at 80 V for 60 min.

### Segregation analysis of transgenic plants

Independent, PCR screened, T_0_ transgenic plants were allowed to set seeds in polyhouse containment facility. Subsequently, the T_1_ generation plants were screened by PCR to analyze the expected transgene segregation ratio (3:1) based on presence or absence of band. Chi-square analysis for transgene inheritance was performed to analyze its segregation pattern. Subsequently, the identification of homozygous transgenic plants was carried out by transgene segregation analysis in T_2_ generation.

### Molecular characterization of transgenic plants

#### Dot blot analysis

The *Hind*III (Fermentas, USA) digested genomic DNA (15 μg) of PCR positive transgenic (T_0_) and WT peanut lines along with plasmid DNA were denatured by boiling (10 min) in 3 M NaOH, followed by the addition of 6X SSC buffer. The denatured DNA was loaded onto nylon membrane (Hybond N^+^, Amersham Pharmacia Biotech, Ireland), wetted in 6X SSC buffer, under low vacuum using DHM-96 dot and slot blotter (SCIE-PLAS, UK). The 528 bp PCR amplified product (using gene-specific primers used for PCR) of plasmid vector was used for the preparation of probe and labeling was done using Biotin DecaLabel DNA Labeling kit (Ferments Life Sciences, USA). Pre-hybridization and hybridization was performed using standard protocols (Sambrook and Russell, 2001) while detection was carried out using the Biotin Chromogenic Detection kit (Ferments Life Sciences, USA).

#### Southern blotting

High quality genomic DNA (30 μg) of transgenic (T_0_) and WT plants was individually isolated using Nucleospin Plant II kit (MN, GmbH). These were then digested with *Hind*III and the genomic DNA of *AtDREB1A* transgenic tobacco (isolated using Nucleospin Plant II kit) was double digested with *Hind*III and *Sac*I (Fermentas, USA) in 1X buffer, separated on 0.8% agarose gel, and transferred to Biodyne plus (0.45 μm) nylon membrane (Pall Life Sciences-Pall corporation, New York, USA) using alkaline transfer buffer (0.4 N NaOH and 1 M NaCl) as per standard protocols (Sambrook and Russell, 2001). The PCR amplified product (528 bp) of *AtDREB1A* gene from transgenic tobacco was used for the preparation of probe, which was then labeled with thermostable alkaline phosphatase, using Amersham Gene Images AlkaPhos Direct Labeling and Detection System (Amersham, GE Healthcare, UK). Hybridization was carried out overnight at 55°C in hybridization buffer and membrane was washed (at 55°C for 15 min) first in primary wash buffer (25 mL) and then in secondary wash buffer at RT. Hybridized membrane was detected using CDP-*Star* chemiluminescent as substrate, and signals were visualized on Amersham Hyperfilm ECL (Amersham, GE Healthcare, UK) after 1 h.

### RNA isolation and reverse transcription PCR (RT-PCR)

Total mRNA was isolated from the leaves of eight Southern positive plants (D1–D8 in T_2_ generation) and the WT (exposed to drought stress) using RNeasy kit (Qiagen, GmbH, Germany). This was subjected to RNase-free DNaseI (Fermentas, USA) digestion and purification (Bhauso et al., 2014b). The RNA was quantified using Nanodrop spectrophotometer (ND-1000, Thermo Scientific, USA) and equal amount of RNA from each sample was used for the two-step RT-PCR reaction. The first-strand of cDNA was synthesized from 500 ng RNA per sample using first strand cDNA synthesis kit (Fermentas, USA) and the product obtained was further used for second-strand amplification using *AtDREB1A* gene specific primers (F: 5′-AAG AAG TTT CGT GAG ACT CG-3′; R: 5′-CTT CTG CCA TAT TAG CCA AC-3′). For internal control, expression of *18S* rRNA gene (F: 5′-GGC TCA AGC CGA TGG AAG T-3′, R: 5′-AGC ACG ACA GGG TTT AAC AAG A-3′).

### Physio-biochemical characterization of transgenic peanuts exposed to progressive soil-moisture deficit stress

The plants of eight transgenic (T_2_) events viz. D1 to D8 were used for the characterization of physio-biochemical and growth-related traits under progressive soil-moisture deficit stress during flowering and pegging stages. The plants were grown in plastic pots (21 × 23 cm) containing 4.5 kg soil mix (1 soil: 1 sand) under containment facility for 45 d until the onset of flowering. The WT plants were divided into two sets as well-watered control (WW), where plants were maintained at about 95% field capacity; and water-stressed control (WS). WS along with the transgenic plants were subjected to drought for a period of 12 d by withdrawal of irrigation, until showing severe wilting symptoms. The experiments were conducted in three replicates. WW plants were used only for phenotypic and growth-related characterization.

The soil-moisture (%) was calculated daily using gravimetric method. The soil-moisture (%) is expressed as the ratio of the mass of water present in the soil sample to the dry-weight of the same soil sample multiplied by 100. The soil-moisture content is calculated from the soil-weight before and after oven drying. Physio-biochemical characterization of all the transgenics and WS were carried out by quantitative estimation of abiotic-stress related parameters such as proline-content, osmotic-potential (OP), relative water content (RWC), electrolytic-leakage (EL), and total chlorophyll content during decreasing soil-moisture regimes at 31, 13, 9, 7, 5% soil-moisture content. Leaves samples, collected from the transgenic and WS plants at the aforesaid soil-moisture contents during drought-stress, were used for further analysis. During the experiment, phenotypic parameters such as wilting of leaves; inhibition of flowering, peg-formation, shoot-length elongation, and development of new-leaf were recorded. At the end of the experiment, 200 ml water was added at an interval of 24 h for recovery of stressed plants up to 3 d and the rate of recovery was visually recorded.

### Proline content

The proline content was estimated according to Bates et al. (1973). The leave samples of both WT and transgenic plants (100 mg) were homogenized using a mortar and pestle in 5 mL sulfosalicylic acid (3%), and centrifuged at 5000 g (10–15 min). The supernatant was collected and diluted to 5 mL with sulfosalicylic acid (3%). Two milliliters each of glacial acetic acid and acid ninhydrin were added to the supernatant (2 mL) and mixed well. The mixture was then boiled for 1 h in a water bath and cooled. Then, for color development, 4 mL toluene was added, mixed well and allowed to stand for 2–3 min. The absorbance of the solution was recorded spectrophotometrically at 520 nm (Epoch microplate spectrophotometer, BioTech, USA). Simultaneously, a blank, containing 2 mL of 3% sulfosalicylic acid was also run. The proline content was calculated as per the proline standard (100 mg/mL in 3% sulfosalicylic acid).

### Osmotic potential

Leaves tissues (100 mg) of transgenics and WS were frozen in liquid nitrogen, followed by thawing in Eppendorf tubes (1.5 mL) at RT for 2 h. It is then centrifuged (at 12,000 rpm for 10 min) to collect the cell-sap. Finally the osmotic potential (osmolality) of cell-sap was measured using Vapor Pressure Osmometer (Model No. 5500; Wescor, USA) (Sarkar et al., 2014).

### Electrolytic leakage (EL)

Fresh leaf discs (1 cm diameter) were washed with distilled water, blotted dried, and incubated in distilled water (25 mL) with continuous shaking for 2 h. Initial electrical conductivity (EC1) was read using pH/EC/TDS Meter (HI991301, HANNA, USA). The leaf discs were then boiled for 30 min in water-bath, followed by cooling at 25°C; and final EC2 was recorded. The percent EL was calculated using the formula (EC1/EC2) × 100% (Wang et al., 2008).

### Relative water content (RWC)

Initial fresh weight (FW) of leaf discs (1 cm diameter) of both transgenics and WS plants were measured. Then the discs were floated in petri-plates containing water for 8 h, and turgid weight (TW) of hydrated leaf-discs were measured. It was then dried in a hot air oven (80°C for 72 h) and weighed till a consistent dry weight (DW) was obtained. RWC was determined using the formula; RWC = (TW–DW)/(FW–DW) × 100 (Barrs and Weatherley, 1962).

### Chlorophyll content

Fifty mg of leaf tissue was kept in 3 mL vial with screw cap (HIMEDIA, India) containing 2 mL dimethylsulfoxide (DMSO), protected from light and incubated in water bath (65°C for 12 h). Absorbance of the extract was read at 645 and 663 nm spectrophotometrically. Chlorophyll content was determined using the following formula:

$$\begin{array}{l} \text{Total chlorophyll }(\text{mg}/\text{g FW})\;=\\ \;[(20.2\times {\text{OD}}_{\text{645}})\;+\;(8.02\;\times \;{\text{OD}}_{\text{663}})]\;\times \text{ V}/(\text{1000 }\times \text{ W}) \end{array}$$

Where, V: volume of extract and W: weight of tissue in g (Hiscox and Israelstam, 1979).

### Quantitative expression of transgene under progressive soil-moisture deficit stress

Quantitative PCR (qPCR) analysis was performed to determine differential expression pattern of *AtDREB1A* gene, after exposing the transgenics at various levels of soil-moisture content (31, 13, 9, 7, and 5%). Total RNA was extracted from the leaf samples and the first strand cDNA was synthesized (as performed for RT-PCR) and qPCR analysis was carried out using a StepOne Real-Time PCR system (Applied Biosystem, USA).

Following first strand cDNA synthesis, the qPCR was carried out in triplicate using gene specific primers (F: 5′-CCT CAG GCG GTG ATT ATA TTC C-3′, R: 5′-ACG ACC CGC CGG TTT C-3′) using QuantiFast SYBR Green PCR Kit (Qiagen, GmbH). The relative quantification of *AtDREB1A* gene was normalized with respect to *18S* rRNA gene as an internal control on qPCR system. Comparative fold expression of transgene was calculated in terms of 2^−ΔΔC_T_^ method (Livak and Schmittgen, 2001). The ΔC_T_ was determined by subtracting *18S* rRNA C_T_ from *AtDREB1A* C_T_ in a given sample. The ΔC_T_ value of “transgenics exposed to 31% soil-moisture content” was used as calibrators. The ΔΔC_T_ value was determined by subtracting the ΔC_T_ of calibrator from ΔC_T_ of transgenics at different soil-moisture regimes. Each reaction was carried out in 20 μL total volume and consist of 1 × SYBR Green Master mix, 20 pmol each primer and 100 ng of diluted cDNA template.

### Evaluation of transgenics for their growth-related traits

At maturity, the transgenics, WS and WW plants were harvested and carefully divided into shoot and root components. The shoot-length, root-length, root-volumes, and number of pods were measured. The dry-weight of roots, shoots, pod, kernel, and total-biomass were determined. Subsequently root: shoot ratio and HI were calculated.

## Statistical analysis

Statistical analysis was carried out with mean value and standard error (SE) of three replicates per analysis and significance of the treatment effects was determined by one-way ANOVA of SPSS 11.0 (Statistical Package For Social Sciences, SPSS Inc., Illinois) at 5% probability level using Tukey test. Correlation coefficient was determined using PAST (PAlaeontological STatistics, ver. 1.89). The qPCR data was analyzed using two-tailed student's *t*-test.

## Results

### Genetic transformation of peanut

**Figure 2:**
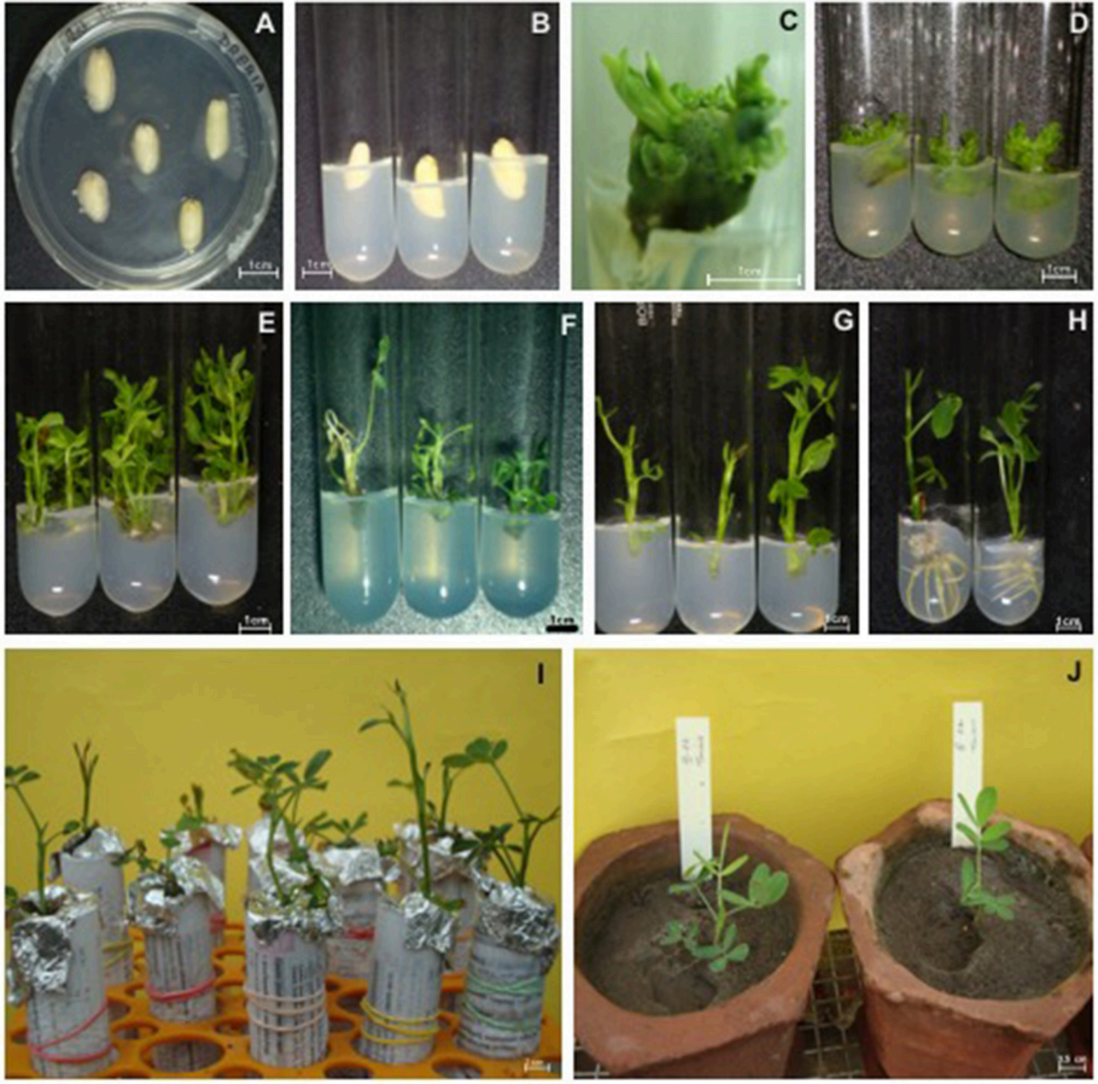
**Genetic transformation and regeneration of peanut from cotyledons. (A–D)** Multiple shoot-buds initiating from co-cultured cotyledonary explants; **(E)** Shoots regeneration; **(F)** Selection in antibiotic amended medium; **(G)** Shoot elongation; **(H)** Rooting; **(I)** Hardening of plantlets in Hoagland's solution; **(J)** Hardened plants grown in pots under containment facility.

Out of 682 *Agrobacterium*-infected explants, 485 responded (71.11% regeneration frequency) and gave rise to 1924 shoots. Further, in the kanamycin (50, 100, and 100 mgL^−1^) amended selection medium, 683 putative transformed shoots (35.5%) were selected (for three cycles of 15 d each) of which, 83.46% produced roots, and 50.52% plantlets were hardened in the containment facility. Co-cultivation of cotyledonary explants with *A. tumefaciens* (strain GV3101) for 3–5 d resulted in 2.49% transformation efficiency (Table 1, Figure 2).

**Table 1:** **Genetic transformation and regeneration of peanut cotyledonary explants from the cv.GG20**.

**Transgene**	**Regeneration (%)**	**Putative transgenics on kanamycin medium (%)**	**Rooting (%)**	**Hardening (%)**	**No of putative transgenic**	**Transformation efficiency (%)**
*AtDREB1A*	71.11	35.50	83.46	50.52	17	2.49

### Molecular characterization

#### Screening of transgenic plants by PCR and dot-blot analysis

**Figure 3:**
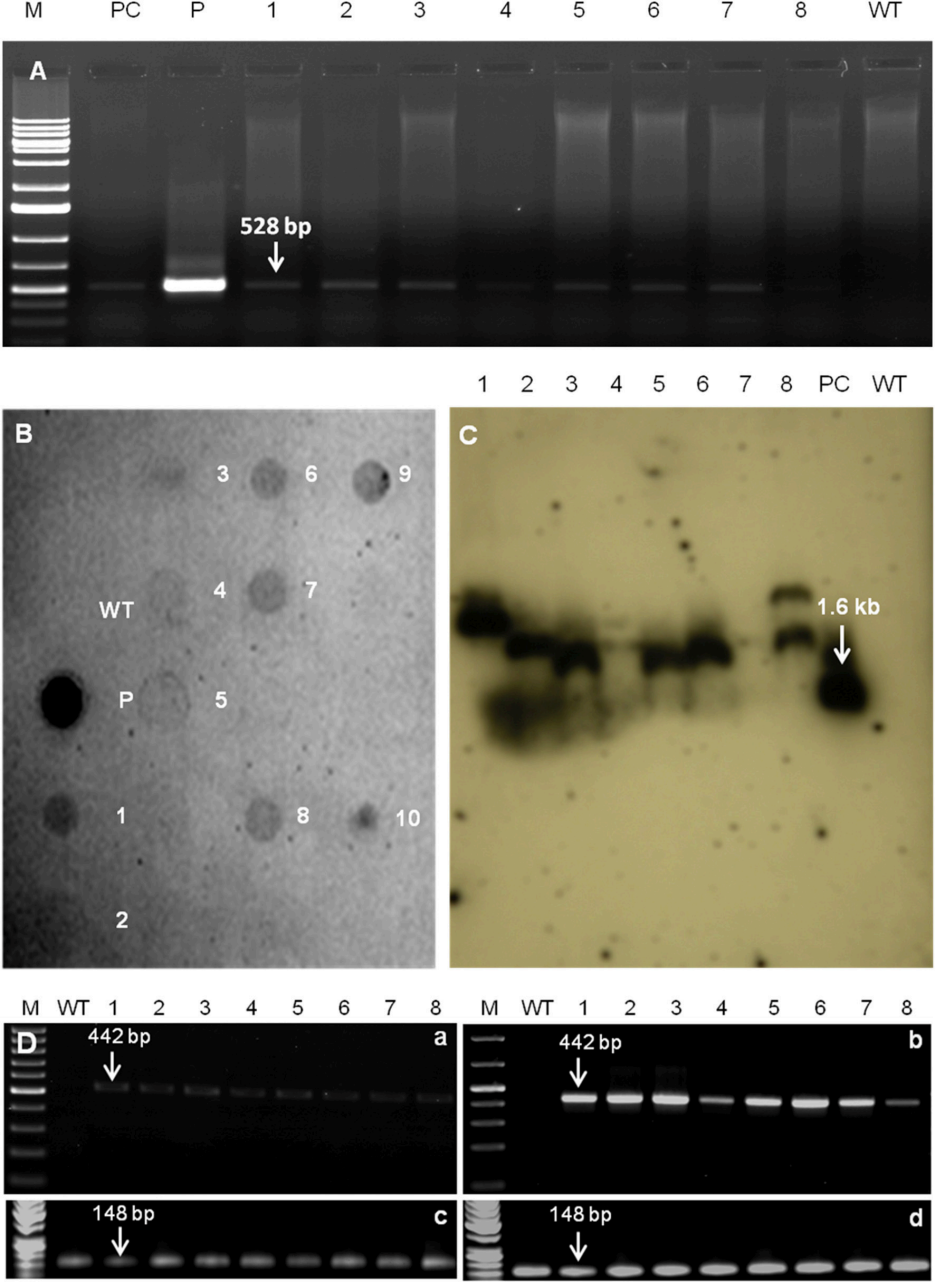
**(A)** PCR analysis of *AtDREB1A* transgenics (T_0_) using gene-specific primers having 528 bp as expected product size. Where lanes, M, marker; PC, positive control (*AtDREB1A* transgenic tobacco); P, plasmid positive control (pCAMBIA2300 plasmid containing *AtDREB1A* gene); 1–8, transgenic plants (D1–D8); WT, genetically non-transformed peanut. **(B)** Dot blot assay of *AtDREB1A* transgenics (T_0_), probed with 528 bp PCR amplified product of *AtDREB1A* coding sequence from plasmid vector pCAMBIA2300 containing *Prd29A*:*AtDREB1A:*3′ *nos* terminator expression cassette. Where, labeled dots, for WT represents *Hind*III digested DNA from genetically non-transformed plant; P, represents plasmid DNA containing *AtDREB1A* expression cassette; numbers 1–10, represents *Hind*III digested *DNA* from 10 putative transgenic lines. **(C)** Southern blot analysis of eight transgenic lines (T_0_). Genomic DNA (30 μg) from individual transgenic peanut plants was digested with *Hind III*, size fractionated on 0.8% agarose gel, transferred to nylon membrane and hybridized with a probe prepared by direct labeling of PCR amplified product (528 bp) of *AtDREB1A* gene with alkaline phosphatase. Where, PC is *Hind*III and *Sac*I digested genomic DNA of *AtDREB1A* transgenic tobacco, WT is *Hind*III digested peanut genomic DNA. **(D)** RT-PCR analysis for *AtDREB1A* gene in eight transgenic and WT plants exposed to 31% **(a,c)** and 5% **(b,d)** soil moisture content using *AtDREB1A* **(a,b)** and *18S* rRNA **(c,d)** genes specific primers. Where, in WT, no expression was observed for *AtDREB1A* gene but expression is recorded for *18S* RNA gene; in all the transgenic plants (D1–D8) expression was recorded for both *AtDREB1A* (442 bp) and *18S* RNA (148 bp) genes.

Screening of 243 putative transgenic peanut plants, grown in containment facility, was carried out by PCR amplification of *AtDREB1A* gene using gene-specific primers (Figure 3A) and 17 putative transgenic (T_0_) plants were identified. Further, Dot-blot confirmed the integration of *AtDREB1A* gene in the genome of ten transgenic (T_0_) plants only (Figure 3B).

#### Southern hybridization

Southern hybridization analysis determined the integration pattern and copy number(s) of *AtDREB1A* gene into the different parts of genome of eight peanut transgenic events. Of 10 dot-blot positive plants, the presence of hybridization signal was recorded in eight T_0_ transgenic plants (D1–D8) (Figure 3C). This analysis confirmed the random integration of single copy *AtDREB1A* transgene in the plants of six transgenic events (D1–D3, D5–D7), while two transgenic plants (D4 and D8) exhibited two copies of transgene in its genome. However, no hybridization signal was observed in the WT.

#### Reverse transcription (RT)-PCR

All the Southern-positive transgenic plants (D1–D8) showed transgene expression in T_2_ generation, as evidenced by RT-PCR. An increase in *AtDREB1A* gene expression was detected at 5% soil-moisture content (drought stress) as compared to 31% soil-moisture content (controlled condition) in transgenic plants, grown in plastic pots under containment facility. Two bands of 442 bp and 148 bp were amplified from the cDNA of different transgenic plants with *AtDREB1A* and *18S* rRNA gene specific primers, respectively (Figure 3D). No transgene expression was observed in the WT.

All RT-PCR positive transgenic plants were subjected to progressive soil-moisture deficit stress for further evaluation of physio-biochemical and growth-related traits. The transcription level of *AtDREB1A* gene was relatively high in D3 and D6 lines compared to that of all other transgenic plants as revealed by RT-PCR analysis, and plants of those two events were used for further qPCR analysis.

#### Transgene inheritance analysis

The transgenic plants having Chi-square value < 3.84 (*p* < 0.05) at 1° of freedom indicated good Mendelian segregation pattern for transgene. In PCR screening, all transgenic plants (D1–D8) in T_1_ generation, showed Mendelian segregation ratio (3:1) of *AtDREB1A* gene (Table 2). These transgenic plants were further advanced to T_2_ generation and based on PCR analysis; homozygous lines in each transgenic event were identified.

**Table 2:** **Segregation analysis of *AtDREB1A* transgene in various transgenic plants in T_1_ generation**.

**Plant No**.	**Total number of T_1_ plants**	**Number PCR positive plants**	**Number of PCR negative plants**	**Test ratio**	**Observed ratio**	**χ^2^**	***P***
D1	8	5	3	3:1	1.67:1^*^	0.667	0.041
D2	8	5	3	3:1	1.67:1^*^	0.667	0.041
D3	7	5	2	3:1	2.5:1^*^	0.048	0.827
D4	6	5	1	3:1	5:1^*^	0.467	0.495
D5	5	4	1	3:1	4:1^*^	0.067	0.796
D6	7	5	2	3:1	2.5:1^*^	0.048	0.827
D7	8	6	2	3:1	3:1^*^	0.000	1.000
D8	7	5	2	3:1	2.5:1^*^	0.048	0.827

Where; D1 to D8 were different transgenic plants. df = 1;

* *Observed ratios were significantly not different from test ratio at P = 0.05*.

### Physio-biochemical analysis of transgenic peanut exposed to progressive soil-moisture deficit stress

#### Proline content

**Figure 4:**
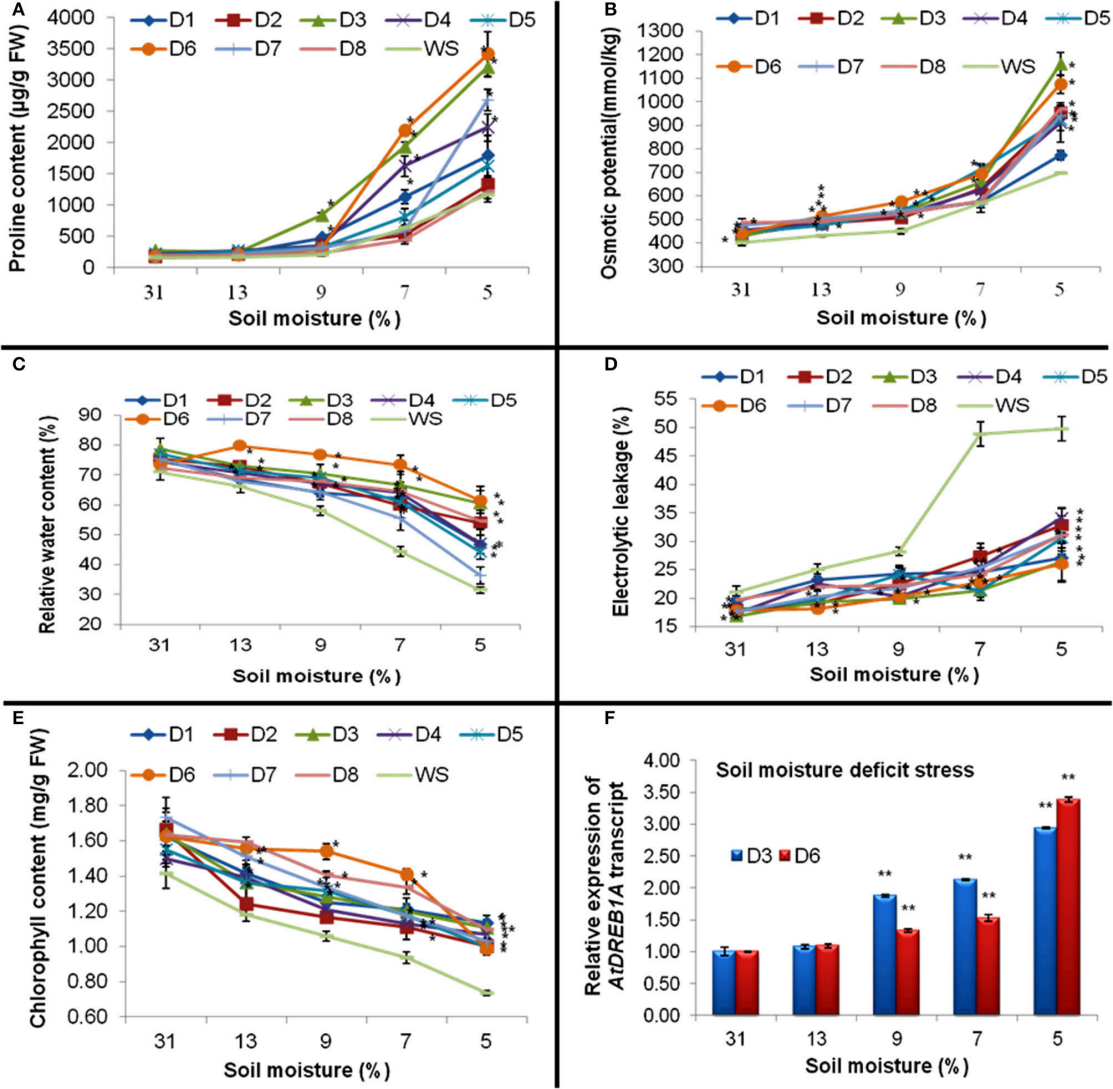
**Effect of progressive decrease in soil-moisture percentage on (A) Proline content, (B) osmotic potential, (C) relative water content (D) electrolytic leakage, and (E) chlorophyll content in the leaves of eight transgenic and WS plants**. Where; D1–D8 were transgenic lines and WS were water-stressed WT plants. Values represent mean activities (*n* = 3) ±SE at *P* = 0.05. (^*^) represent values significantly different at *P* ≤ 0.05. **(F)** Expression of *AtDREB1A* transcript as analyzed by quantitative PCR in two transgenic plants at different soil moisture regimes. The level of *AtDREB1A* transcript in transgenic plants was normalized with reference to *18S* rRNA taken as an internal control. Data represent means of three replicates. Bars denote fold expression as compared to the expression level at 0 day ± SD. Two-tailed Student's *t*-test was used to determine highly significant (^**^P ≤ 0.01).

Up to 13% of the soil-moisture content, no significant difference in proline content in WS and transgenic plants were recorded. However, at 7% soil-moisture, plants of five transgenic events (D1, D3, D4, D5, and D6) showed very high accumulation of proline; while plants of three transgenic events (D2, D7, and D8) expressed proline content even less than WS. Moreover, at 5% soil moisture, all transgenic plants accumulated more proline than WS, of which D3 and D6 showed maximum proline accumulation to the tune of 2.75 and 2.93 folds respectively (Figure 4A).

#### Osmotic potential

At 31% soil-moisture, not much difference in osmotic potential was recorded in WS and all transgenic plants. However, across other soil-moisture regimes (starting from 13%), all transgenic plants exhibited significantly better osmotic potential compared to WS. When observed at 5% soil-moisture, two transgenics D3 and D6 prominently showed better osmotic potential over other transgenic plants and WS (Figure 4B). However, a positive correlation was detected between proline content and osmotic potential (*r* = 0.58; Table 3) in all transgenics and WS under drought stress.

**Table 3:** **Correlation coefficient (*r*) between growth-related and physio-biochemical parameters of transgenic and WT plants exposed to 5% soil-moisture content**.

	**RW**	**SW**	**PW**	**TB**	**HI**	**RSR**	**PR**	**OP**	**RWC**	**CH**	**EC**
RW	1.00										
SW	0.96^**^	1.00									
PW	0.91^**^	0.87^**^	1.00								
TB	0.98^**^	0.98^**^	0.95^**^	1.00							
HI	−0.18	−0.31	0.15	−0.14	1.00						
R:S	0.21	−0.05	0.23	0.09	0.53^**^	1.00					
PR	0.57^**^	0.41^*^	0.56^**^	0.51^**^	0.21	0.61^**^	1.00				
OP	0.69^**^	0.59^**^	0.67^**^	0.65^**^	0.13	0.43^*^	0.58^**^	1.00			
RWC	0.52^**^	0.48^**^	0.58^**^	0.53^**^	0.20	0.18	0.41^*^	0.78^**^	1.00		
CH	0.33	0.22	0.41^*^	0.31	0.51^**^	0.48^**^	0.38^*^	0.41^*^	0.53^**^	1.00	
EC	−0.47^**^	−0.37	−0.61^**^	−0.48^**^	−0.52^**^	−0.43^*^	−0.73^**^	−0.50^**^	−0.66^**^	−0.58^**^	1.00

For each parameter, average values of three AtDREB1A transgenic peanut from each plant along with WS were used. Where, WS, water-stressed; WT, wild-type; RW, root-weight; SW, shoot-weight; PW, pod-weight; TB, total biomass; HI, harvest index; RSR, root:shoot ratio; PR, Proline; OP, Osmotic potential; RWC, Relative water content; EC, Electrolytic leakage; CH, Chlorophyll content. Where, ^*^ and ^**^ indicates significant and highly significant correlation at ^*^P ≤ 0.05, and ^**^P ≤ 0.01 respectively.

#### Relative water content

From 13 to 7% of soil-moisture regimes, significantly higher RWC was recorded for all the transgenic plants over WS. But, at 5% soil moisture, plants of D3 and D6 transgenic events witnessed nearly 40% more RWC than WS and were found better than other transgenics (Figure 4C). A positive correlation was noticed between osmotic potential and RWC (*r* = 0.78) in transgenic plants and WS under drought-stress (Table 3).

#### Electrolytic leakage

Although at 31 and 13% soil-moisture content, there were some differences in EL between WS and transgenic plants; but at 5 and 7% of soil-moisture, all transgenic plants witnessed significantly less EL than WS. Moreover, at 5% soil-moisture, D3 and D6 plants showed significantly less EL over other transgenic plants and WS (Figure 4D). However, a significant negative correlation was observed between RWC and EC (*r* = −0.66) in all transgenic plants and WS under drought stress (Table 3). Furthermore, higher proline accumulation was found negatively correlated with membrane injury or EL in transgenics and WS under drought stress (*r* = −0.73, Table 3).

#### Total chlorophyll content

Although, at various levels of moisture-deficit stress; all the transgenic plants were found to have significantly more chlorophyll content than WS. Moreover, at 5% soil-moisture, plants of D1, D3, and D8 transgenic events retained relatively more chlorophyll content than WS and other transgenic plants (Figure 4E).

#### qPCR analysis of transgenic plants exposed to progressive soil-moisture deficit stress

Of eight transgenic events, homozygous D3 and D6 plants showed maximum transgene expression in RT-PCR assay. Further, improved physio-biochemical traits under drought stress were used for the qPCR analysis. At 9% soil-moisture, an increase in the transgene expression to the tune of 1.88 and 1.33 folds and at 5% of soil-moisture, an increase of 2.94 and 3.39 folds were recorded in plants of D3 and D6 events respectively (Figure 4F). Under progressive soil-moisture deficit stress, *AtDREB1A* gene expression in both the transgenic events were found positively correlated with the improved physio-biochemical parameters like proline content (*r* = 0.66), osmotic potential (*r* = 0.63), EL (*r* = 0.52); while negative correlation was observed with chlorophyll content (*r* = −0.73), RWC (*r* = −0.60) (Table 4).

**Table 4:** **Correlation coefficient (r) between *AtDREB1A* gene expression and different physio-biochemical parameters in two transgenic plants (D3 and D6) under progressive soil-moisture deficit stress**.

	**PR**	**OP**	**RWC**	**EC**	**CH**	***AtDREB1A***
PR	1.00					
OP	0.91^**^	1.00				
RWC	−0.70^**^	−0.66^**^	1.00			
EC	0.69^**^	0.76^**^	−0.71^**^	1.00		
CH	−0.75^**^	−0.74^**^	0.73^**^	−0.67^**^	1.00	
*AtDREB1A*	0.66^**^	0.63^**^	−0.60^**^	0.52^**^	−0.73^**^	1.00

*For each parameter, average values of three AtDREB1A transgenic peanut from each plant were used (Refer to Table 3)*.

#### Visual observations under progressive soil-moisture deficit stress

All the transgenic plants showed improved phenotypic traits over WS during soil-moisture deficit stress and WS exhibited wilting of leaves at 9% soil-moisture, while transgenic plants showed wilting, only when soil-moisture becomes < 7%. Moreover, at 5% soil-moisture, WS showed severe wilting compared to transgenics (data not shown). Upon withdrawal of stress, transgenic plants showed speedy recovery than WS.

#### Evaluation growth-related parameters

Growth-related parameters like shoot, root, pod and kernel traits were recorded for all the transgenics and WT (WS and WW). Among different transgenics and WS, D3 line was found significantly better for shoot-length and shoot-weight; however, it was significantly higher for WW than all transgenics and WS (*P* < 0.05) (Table 5). Transgenic plants of D3 and D6 events, exhibited significantly better root-length, root-weight, and root-volume than WS and WW; which might had helped in improving its WUE under drought-stress. Moreover, negative impact of drought-stress was recorded for pod-biomass more prominently in WS compared to transgenic and WW lines. Likewise, kernel-weight, total-biomass and HI of all transgenics and WW were higher or at par with WS lines.

**Table 5:** **Growth-related parameters of transgenic and WT (WS and WW) lines grown under soil-moisture deficit stress**.

**Plant ID**	**Root-weight (g)**	**Shoot-weight (g)**	**Pod-weight (g)**	**Total-biomass (g)**	**HI**	**Rooot: Shoot ratio**	**Kernel-weight (g)**	**Root-volume (mL)**	**Shoot-length(cm)**	**Root-length(cm)**
D1	1.51± 0.22^cd^	4.37± 0.42^bc^	3.78± 0.51^b^	9.67± 1.17^bc^	0.39± 0.01^b^	0.34± 0.02^c^	3.08± 0.15^b^	4.83± 0.60^cd^	15.02± 0.78^c^	18.43± 0.41^bc^
D2	1.61± 0.39^c^	4.50± 0.99^bc^	3.55± 0.60^b^	9.65± 1.97^bc^	0.37± 0.01^b^	0.35± 0.01^c^	2.45± 0.52^b^	4.9± 0.31^cd^	16.38± 2.16^c^	21.00± 0.68^bc^
D3	3.82± 0.22^a^	9.58± 0.92^a^	6.06± 0.48^ab^	19.45± 1.60^a^	0.31± 0.01^c^	0.40± 0.02^bc^	3.90± 0.25^ab^	13.93± 0.47^a^	23.38± 1.17^a^	28.30± 0.65^ab^
D4	1.01± 0.04^d^	2.38± 0.24^c^	2.03± 0.16^c^	5.42± 0.41^d^	0.38± 0.01^b^	0.43± 0.03^b^	1.47± 0.05^c^	3.5± 0.76^d^	13.70± 0.31^c^	16.33± 0.73^c^
D5	1.76± 0.10^bc^	4.69± 0.21^bc^	4.05± 0.17^b^	10.51± 0.40^bc^	0.39± 0.01^b^	0.38± 0.01^bc^	3.23± 0.64^b^	5.00± 0.29^cd^	15.20± 0.20^c^	23.37± 5.47^b^
D6	2.24± 0.06^b^	5.18± 0.09^bc^	5.06± 0.27^ab^	12.48± 0.22^b^	0.40± 0.01^b^	0.43± 0.02^b^	3.74± 0.43^ab^	10.43± 0.30^b^	18.57± 0.23^bc^	29.43± 1.23^a^
D7	1.82± 0.09^bc^	3.67± 0.24^c^	3.76± 0.16^b^	9.25± 0.48^c^	0.41± 0.01^b^	0.50± 0.02^a^	2.62± 0.08^b^	5.47± 0.64^c^	15.90± 0.32^c^	22.80± 0.93^b^
D8	1.98± 0.13^bc^	4.73± 0.52^bc^	4.24± 0.13^b^	10.96± 0.71^bc^	0.39± 0.02^b^	0.42± 0.03^b^	3.34± 0.16^b^	5.72± 0.97^c^	16.33± 1.19^c^	21.90± 2.12^bc^
WS	0.94± 0.02^d^	2.92± 0.10^c^	1.65± 0.14^c^	5.51± 0.18^d^	0.30± 0.02^c^	0.32± 0.02^c^	0.81± 0.03^c^	2.60± 0.38^d^	16.40± 0.46^c^	17.60± 0.55^bc^
WW	0.55± 0.05^d^	5.28± 0.09^b^	5.40± 0.22^ab^	11.23± 0.14^bc^	0.48± 0.01^a^	0.10± 0.01^d^	4.34± 0.20^a^	2.00± 0.29^d^	20.05± 0.31^b^	15.96± 0.87^c^

*Where, D1 to D8, transgenic plants; WW and WS, well-watered and water-stressed WT respectively. The data are mean of three replicates ±SE; Means followed by the same lower case letters within a column are not significantly different (P ≤ 0.05)*.

Similarly, root: shoot ratio of all the transgenics were higher or at par with WS lines under soil-moisture deficit stress (Table 5). Chlorophyll content showed positive correlation with pod-weight (*r* = 0.41) and HI (*r* = 0.51); and it also witnessed positive correlation with improved root: shoot ratio in transgenic and WS (*r* = 0.48) under drought stress (Table 3). Both transgenics and WS showed improved root: shoot ratio which was found positively correlated with HI (*r* = 0.53; Table 3) under drought stress. Overall, transgenic plants D3 and D6 showed better root-length, root-volume, root-weight, pod-weight, total-biomass, kernel-weight and root: shoot ratio than WS plants, indicating the best agronomic performers across all eight transgenic plants under drought stress (Table 5).

## Discussion

This investigation deals with the development of eight *AtDREB1A* transgenic events (D1–D8) and its characterization at reproductive growth stage, for drought-stress using progressive soil-moisture deficit stress, having advantage of mimicking the real-field conditions. Whereas, the results published earlier (Sarkar et al., 2014) is part of the ongoing work in our lab, which reports the characterization of only three transgenic events (D1–D3) at seedling and vegetative-growth stages for both drought and salinity stresses using different levels of Polyethylene glycol (PEG) and NaCl respectively, under hydroponic conditions.

In this investigation, *Agrobacterium*-mediated genetic transformation, successfully transformed the cotyledonary explants of peanut cv. GG20 with *AtDREB1A* gene and shoots were regenerated following the protocols previously reported in peanut by our group (Radhakrishnan et al., 2000; Mehta et al., 2013; Bhauso et al., 2014b). Further, cotyledon have been used as explants, as it showed better transformation efficiency, compared to immature peanut leaves as reported by Mehta et al. (2013). The selection scheme has identified the putatively transformed shoots in kanamycin amended selection medium based on visual observations, and genetically non-transformed shoots have been eliminated (Bhatnagar-Mathur et al., 2007).

This investigation produced transgenic peanut (cv. GG20) with *AtDREB1A* transgene, under the regulatory mechanism of stress inducible *rd29A* promoter, with 2.49% transformation efficiency. The regeneration of multiple shoots from cotyledonary explants was 71.11%, which validated the reports on genetic transformation of peanut, where it ranged from 60.4 to 84.3% (Bhatnagar-Mathur et al., 2007; Mehta et al., 2013). However, certain other reports showed varying transformation efficiency in different genotypes like 5.18% in cv. GG20 (Bhauso et al., 2014b); 16.5% in cv. K134, 34% in cv. K6 (Mehta et al., 2013) and 70% in cv. JL24 (Bhatnagar-Mathur et al., 2007). It means transformation efficiency is genotype dependent in peanut, as also suggested by Krishna et al. (2015).

Most of the tissue culture raised transgenic plants (T_0_) produced less number of seeds (5–8), as also reported on *cry1EC* transgenic peanut (Tiwari et al., 2008), and all the transgenic plants (T_1_) showed Mendelian segregation ratio (3:1). Further, PCR based screening was used for the progeny advancement, thereby helping in analyzing the transgene inheritance pattern appropriately. Southern analysis showed both, stable integration and copy number(s) of transgene in the genome of individual transgenic plants (T_0_). No phenotypic alteration in any transgenic plant was recorded, compared to WT under controlled condition; indicating the absence of any metabolic load in transgenics, no disruption of any functional endogenous gene(s), and tightly regulated expression of *AtDREB1A* transgene under drought-stress as also reported in earlier studies (Vivek et al., 2013; Sarkar et al., 2014).

RT-PCR analysis showed elevated levels of transgene-expression in eight homozygous transgenic plants (T_2_) under drought-stress (Figure 3D), indicating a positive relationship between transgene expression and stress-tolerance as also observed in case of *mtlD* transgenic peanut (Bhauso et al., 2014b). Furthermore, even under controlled conditions, RT-PCR showed some basal level of transgene expression in all transgenic plants, which might be responsible for the relatively better physio-biochemical parameters of transgenics than WT. Similarly, basal level of transgene expression were also observed in *AtDREB1A* transgenic *Arabidopsis* (Kasuga et al., 1999) and tomato lines (Rai et al., 2013), under unstressed condition. Although, very minute activity of transgene expression was detected under unstressed conditions, but *rd29A* promoter is still considered stress-inducible due to the rapid induction and higher level of *AtDREB1A* gene expression under stress, compared to the 35S promoter (Sarkar et al., 2014).

The PCR, dot-blot, southern-blot and RT-PCR results were effectively utilized for the identification and selection of transformed plants of eight transgenic events. Further, confirmed transgenic plants (T_2_), were then used for the evaluation of physio-biochemical and growth-related parameters. The drought-tolerance evaluation of transgenic plants, during appropriate developmental stage, like initiation of flowering (under field like conditions) is crucial for the identification of better transgenic lines (Bhatnagar-Mathur et al., 2010; Ravikumar et al., 2014). The scheme of progressive soil-moisture deficit stress, followed in this study, for the imposition of drought-stress, has the advantage of mimicking the real-field conditions (Bhatnagar-Mathur et al., 2008; Anbazhagan et al., 2015).

Accumulation of various osmolytes in different concentrations, under drought stress is known to result in the osmotic adjustments, which in turn impart tolerance to the plants (Macková et al., 2013; Ravikumar et al., 2014; Nahar et al., 2015). Proline is one such important osmolytes which gets accumulated as the first line of response, when plants were exposed to drought stress (Ghobadi et al., 2013; dosSantos et al., 2013; Bhauso et al., 2014a). In this study too, proline levels has been increased profoundly as a function of decreasing soil-moisture content. Similar observation were also reported for *AtNDPK2* transgenic alfalfa (Wang et al., 2014), *mtlD* transgenic peanut (Bhauso et al., 2014a,b) and *AtDREB1A* transgenic peanut under both drought and salinity stresses (Sarkar et al., 2014).

A gradual increase in osmotic-potential was recorded, after imposition of progressive soil-moisture deficit stress, across all the transgenic, and WS peanut lines. However, transgenics exhibited better osmotic adjustment than WS in both the investigations (Sarkar et al., 2014). Increase in osmotic potential, by way of proline accumulation is known to be a vital plant defense mechanism to face environmental constraints (Pruthvi et al., 2014). Positive correlation between proline content and osmotic potential (Table 3) reiterated the fact that free proline accumulation might be responsible for better osmotic adjustments (Hayat et al., 2012).

RWC, often used to determine the water retention capacity and drought tolerance, reflects metabolic activities of plant tissues (Ravikumar et al., 2014). The transgenic peanut plants showed more RWC, compared to WS, under drought stress. It is assumed that the improved membrane integrity in transgenics over WS resulted in controlled water-efflux as indicated by better RWC under drought stress. Similar reports were also observed in *LEA Rab28* transgenic maize (Amara et al., 2013) and *AtNDPK2* transgenic alfalfa (Wang et al., 2014) under drought stress. Positive and negative correlation of RWC with osmotic potential and EC respectively, in all the transgenic and WS (Table 3) peanut lines supports the analogy that improved osmotic adjustment reflects higher water retention capacity, lower rate of water loss, as well as higher WUE (Mao et al., 2012).

Abiotic stress imparts detrimental effects on membrane integrity, as also reflected by higher EL. Moreover, the leaking ions are equally vital for the proper functioning of cell (Khare et al., 2010). A gradual increase in EL along with progressive decline in soil-moisture content, across all the transgenic and WS peanut lines was recorded. But, transgenic peanut lines showed relatively less EL compared to WS, as also reported by Gao et al. (2013) on transgenic *TaLEA* poplar under drought-stress. Similarly, Xu et al. (2014) also reported that *MaPIP1;1* transgene insertion in *Arabidopsis* genome conferred enhanced drought-stress tolerance by reducing membrane injury and maintaining osmotic balance in transgenics. A negative correlation was observed between the proline content and EL (Table 3), which means, proline accumulation might be responsible for lesser EL in both transgenics and WS; but, EL was quite less in transgenics than WS (Hayat et al., 2012).

The activation of stress-response pathway of any plant is highly energy-demanding mechanism; therefore, protection of photosynthetic machinery contributes to its ability to withstand the stress. Extensive reactive oxygen species (ROS) were generated by the drought stress which is detrimental to the photosynthetic machinery including D1 protein of photosystem II (PSII) and total-chlorophyll content (Wang et al., 2008; Macková et al., 2013). Under decreasing soil-moisture regime, transgenics peanut plants showed significantly less reduction in total chlorophyll content than WS, which is in agreement with various other reports (Amara et al., 2013; Liu et al., 2014). The chlorophyll retention capacity might have resulted in improved pod- and kernel- weight of transgenic than WS peanut lines, as also reported for *AtDREB1A* transgenic rice (Ravikumar et al., 2014).

qPCR analysis showed a consistent increase of around three-fold in the expression of *AtDREB1A* gene in transgenic peanut (D3 and D6) with progressive decrease in soil-moisture content (Figure 4F). Similarly, in our previous study, more than two-fold increase in *AtDREB1A* gene expression was observed within 2 h of drought (20% PEG) and salinity (200 mM NaCl) stress imposition (Sarkar et al., 2014). Furthermore, soil-moisture contents had direct impacts on the level of transgene expression in the leaves of transgenic plants. It means these transgenic plants could be used for the peanut genetic improvement programmes for drought tolerance.

Under stressed conditions, transgenic plants showed delay in the wilting of leaves; and speedy recovery of transgenics over WS after withdrawal of stress. This could be supported by the fact that there is a consistent increased in expression of *AtDREB1A* gene in transgenics across various soil-moisture regimes, as also reported in other transgenic crops expressing various TFs such as *LbDREB* (Ban et al., 2011); *AtDREB1A* (Bhatnagar-Mathur et al., 2014); *AtDREB2A, AtHB7*, and *AtABF3* (Pruthvi et al., 2014).

Our earlier studies on three *AtDREB1A* transgenic peanut lines (D1, D2, and D3) also showed better tolerance of transgenics to both PEG induced drought and NaCl induced salinity stresses, and its rapid recovery upon withdrawal of stress, compared to WT (Sarkar et al., 2014). All the transgenics exhibited improved water retention capacity compared to WS peanut lines, which could be due to the regulation of stomatal behavior and transpiration under stress (Datta et al., 2012; Anbazhagan et al., 2015). However, it needs further experimental confirmation for our transgenic plants.

Correlations were elucidated between *AtDREB1A* gene-expression and various physio-biochemical parameters in response to drought-stress (Table 4). Significant positive correlation between *AtDREB1A* gene-expression with proline content and osmotic potential; signifies the fact that transgene expression could be one of the reasons for improved osmotic adjustments (Figures 4A,B; Table 4). A negative correlation between transgene expression with RWC and chlorophyll content could be attributed in part to the enhanced water and chlorophyll retention capacity of transgenics (Table 4). Positive relationship was observed between transgene expression and EC under drought stress (*r* = 0.52) (Figure 4D; Table 4). This means, enhanced tolerance in transgenic peanut was associated with stress inducible expression of *AtDREB1A* gene, as also reported in *AtDREB1A* transgenic tomato (Rai et al., 2013).

Growth-related parameters indicated that, transgenics performed better than WS and showed full potential growth and yield, at severe drought-stress (up to 5% soil moisture) during reproductive growth stages (Table 5). Based on phenotypic response, physio-biochemical characterization, and transgene expression analysis, it could be inferred that at ≤ 9% soil-moisture content, transgenics made better use of stress-responsive mechanism than WS, which could be due to heterologous expression of *AtDREB1A* gene. Differential improvement in various growth-related traits including shoot-, root-, pod-, and kernel-traits at maturity was also observed in our earlier report (Sarkar et al., 2014).

Significantly, improved root-traits like root: shoot ratio, root-volume of transgenic plants over WS under drought stress, could be due to better *AtDREB1A* gene expression in root system compared to leaf tissues as also reported by Ban et al. (2011). Improved root-architecture like profuse and deeper rooting system in transgenics might be the reason for enhanced water uptake from deeper soil layer, resulting in improved yield, as also observed in *AtDREB1A* transgenic peanut by Vadez et al. (2013). Furthermore, most of the transgenic plants showed improved total-biomass, pod-weight, kernel-weight and HI over WS under drought stress.

Transgenics also showed better partitioning of total-biomass, more toward roots and pods, than shoots. This might have resulted in higher root: shoot ratio, pod-weight, and HI in transgenics, compared to WS. Moreover, higher water-uptake capacity of transgenic peanut lines was considered as a function of increased root: shoot ratio under drought stress (Vadez et al., 2007). Positive correlation was found between root: shoot ratio and HI under drought stress and transgenics showed better improvement for these traits than WS (Tables 3, 5). It means better root: shoot ratio might have contributed to the enhancement in HI more prominently in transgenics than WS. Similarly, positive relationship between improved root-traits and productivity was observed in *AtDREB1A* transgenic peanut under soil-moisture deficit stress (Vadez et al., 2007; Jagana et al., 2012; Bhatnagar-Mathur et al., 2014).

Moreover, transgenic plants showed improved root: shoot ratio and root-traits over WS which might have resulted in better access of roots to the deeper soil-layers for improved water uptake (Bhatnagar-Mathur et al., 2010; Anbazhagan et al., 2015). Based on correlation analysis (Table 3), it could be inferred that an enhanced (denser and deeper) root-system in transgenics might be responsible for improved accumulation of ions (especially Mg^2+^) from soil which seems to also have a positive role in the chlorophyll retention capacity under drought stress (Werner et al., 2010; Macková et al., 2013).

Furthermore, *AtDREB1A* transgenic rice lines derived from a single plant also showed difference in stress tolerance, physiological, and growth parameters under drought stress (Ravikumar et al., 2014). Improved physio-biochemical and growth-related parameters of the transgenic peanut plants, might be either due to the activation of an array of drought inducible genes or devising some sort of conservative growth pattern as drought tolerance/avoidance mechanism compared to WS (Bhatnagar-Mathur et al., 2008; Turyagyenda et al., 2013; Ravikumar et al., 2014). Thus, both investigations showed that, *AtDREB1A* transgenic peanut events developed in our lab revealed abiotic-stress tolerance.

## Conclusions

A premium peanut variety (GG20) of India, with high marketability, but not tolerant to drought was used for the development of drought tolerant *AtDREB1A* transgenics. Both, present investigation and our earlier report (Sarkar et al., 2014) demonstrated better tolerance of *AtDREB1A* transgenic plants to drought, not only at seedling and reproductive growth stages, but also at maturity level. Out of eight transgenic events (viz. D1–D8), two D3 and D6, clearly showed much improved drought tolerance under progressive soil-moisture deficit stress. A strong association was recorded between *AtDREB1A* gene induction and drought-stress tolerance in the transgenic plants. It seems that the enhancement of various growth-related traits could be due to the prompt and tightly regulated expression of *AtDREB1A* gene, driven by stress inducible *rd29A* promoter, and its subsequent participation in signaling cascade followed by up-regulation of various stress-inducible, downstream endogenous genes (Kasuga et al., 1999; Ravikumar et al., 2014), which eventually have resulted in drought-stress tolerance. This study could be a valuable attempt for application of transgenic peanut in agriculture, and subsequently in trade and commerce. Although, a few transgenic peanut plants having enhanced drought tolerance have been developed by various groups of researchers; but so far, no commercial varieties have been released (Bhauso et al., 2014b) from any part of the World. Thus, the transgenic plants characterized in this study, can be used further for the contained multi-year, multi-location trials under real-field conditions. Subsequently, the best one(s) can be used in the breeding programmes either as valuable pre-breeding resources or directly as enhanced drought-tolerant genotype for commercial cultivation.

## Author contributions

RT: Conceived the experiment and planned. TS: Executed the experiments and recorded data. AK: Assisted in carrying out the laboratory work. GM: Prepared the manuscript. JD: Assisted in carrying out the laboratory work.

### Conflict of interest statement

The authors declare that the research was conducted in the absence of any commercial or financial relationships that could be construed as a potential conflict of interest.

## Abbreviations

ABF3: ABRE binding factor
HB7: Homeodomain-leucine zipper
DREB2A: Dehydration responsive element-binding factor.
